# Exhaled Breath Condensate: A Promising Source for Biomarkers of Lung Disease

**DOI:** 10.1100/2012/217518

**Published:** 2012-12-17

**Authors:** Yan Liang, Samantha M. Yeligar, Lou Ann S. Brown

**Affiliations:** ^1^Division of Neonatal-Perinatal Medicine, Department of Pediatrics, Emory University and Emory+Children's Healthcare of Atlanta Center for Developmental Lung Biology, Atlanta, GA 30322, USA; ^2^Department of Medicine, Atlanta Veterans' Affairs and Emory University Medical Centers, Decatur, GA 30033, USA

## Abstract

Exhaled breath condensate (EBC) has been increasingly studied as a noninvasive research method for sampling the alveolar and airway space and is recognized as a promising source of biomarkers of lung diseases. Substances measured in EBC include oxidative stress and inflammatory mediators, such as arachidonic acid derivatives, reactive oxygen/nitrogen species, reduced and oxidized glutathione, and inflammatory cytokines. Although EBC has great potential as a source of biomarkers in many lung diseases, the low concentrations of compounds within the EBC present challenges in sample collection and analysis. Although EBC is viewed as a noninvasive method for sampling airway lining fluid (ALF), validation is necessary to confirm that EBC truly represents the ALF. Likewise, a dilution factor for the EBC is needed in order to compare across subjects and determine changes in the ALF. The aims of this paper are to address the characteristics of EBC; strategies to standardize EBC sample collection and review available analytical techniques for EBC analysis.

## 1. General Characteristics of EBC 

**Figure 1:**
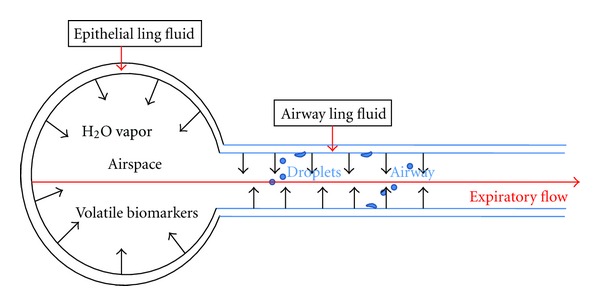
Nonvolatile and volatile components in EBC. Water vapor is rapidly diffused from the lining fluid on the surface of the airway (bronchi) and airspace (alveolar) into the expiratory flow. Droplets (nonvolatile biomarker) formation in the lung is largely from the lining fluid of the airway where turbulence is encountered. Respiratory gases (volatile biomarkers) are from both airspace and airway, and more soluble vapors are typically greater in the airway [5, 67].

Exhaled breath condensate (EBC) is collected from exhaled breath, usually through a refrigerated device [1–4]. During exhalation, volatile molecules and water evaporation directly diffuse as gases from the lining fluid covering airspaces (e.g., alveoli), airways (e.g., bronchi), and the mouth. These gases are then collected into the expiratory air flow. Laser particle counting revealed that micron- and submicron-sized droplet particles are formed in the exhaled breath. Such particles serve as the only evidence of nonvolatile components in the EBC [4]. Yet, the nature and source of exhaled particles/droplets in the EBC matrix are not fully understood. Droplet formation within the lungs during exhalation is largely in the airways where turbulence is encountered (Figure 1). In addition, energy to overcome surface tension during inspiration may also apply to the airway and alveoli, potentially creating exhalable particles. However, the major source for nonvolatile components in the EBC is believed to be the airway lining fluid (ALF) [5, 6]. The main components of EBC include condensed water vapor, volatile molecules (such as nitric oxide, carbon monoxide, and hydrocarbons), and nonvolatile molecules (such as urea, GSH, leukotrienes, prostanoids, and cytokines) [1, 3]. 

## 2. Aspects of EBC Sample Collection

EBC sampling has great advantages over bronchoalveolar lavage (BAL) because it is noninvasive. However, components of the ALF are highly diluted in the EBC and are mixed with compounds from the mucus layer of the airway. Reproducibility is a major concern in EBC sample collection [1]. During EBC collection, tidal breathing is recommended [7]. With tidal breathing, the volume of air that is inhaled or exhaled is included in a single breath. This normal resting breathing pattern generates a reproducible volume of EBC and sampling of the ALF. However, EBC volume is not the only variable but different mediators in EBC can contribute to greater variability than volume. The causes of this variability have yet to be fully investigated but include the varied dilution effects for different nonvolatiles and technique sensitivity needed for these nonvolatiles. Although EBC dilution is relevant for nonvolatile constituents of EBC, this is not relevant for volatile components. Currently the estimated dilution factor is determined using nonvolatile molecules which have similar concentrations in ALF and plasma. Dilution factors that have been used include urea, cations, total protein concentration, or the conductivity of lyophilized EBC [2, 6, 8]. The dilution (*D*) of an interested nonvolatile biomarker can be calculated as shown below, using urea as the standard indicator:

(1) $$\begin{array}{c} {\left[\text{nonvolatile}\right]}_{\text{ALF}}=D\times {\left[\text{nonvolatile}\right]}_{\text{EBC}},\\ \text{where}\;\;D=\frac{{\left[\text{Urea}\right]}_{\text{ALF}}}{{\left[\text{Urea}\right]}_{\text{EBC}}}=\frac{{\left[\text{Urea}\right]}_{\text{plasma}}}{{\left[\text{Urea}\right]}_{\text{EBC}}}. \end{array}$$

However, it needs to point out that the dilution of these nonvolatile biomarkers by water vapor can vary dramatically, and to date, there is no gold standard for assessing the dilution of ALF biomarkers in the EBC [1, 6, 9, 10]. 

Clearly, the interest in EBC relies on its ease in sample collection. However, sufficient and reproducible techniques are needed in EBC sampling. Recommendations for EBC sample collections are well documented and accepted among EBC researchers. In brief, precondensation conditions, such as ambient air and environment temperature, should be recorded; the condenser's design, material, surface area and cooling temperature should be well adjusted; the subject's conditions, such as medications, tobacco smoking, food and drinks, exercise, et al., can have significant effects on EBC collection and should be recoded and adjusted. Detailed recommendation conditions for EBC collection have been described in [1, 4, 11, 12]. 

## 3. Measurements of Mediators in EBC

As described above, EBC components are classified into two categories: volatile and nonvolatile. Guidelines and recommendations to measure EBC substances are useful in standardizing measurements and further develop new techniques. The following issues are generally considered when measuring substances in EBC [1, 4, 11, 13]. (1) The cooling temperature range should be specified. Colder condensation temperature is usually better for unstable EBC components; however, colder temperature may reduce the amount of volatiles because they are more readily absorbed into the liquid phase. (2) Sublimation of the volatiles into the airspace should be considered when using frozen storage for EBC samples. (3) Each substance of interest should be studied in detail to control for the potential effects of duration of EBC collection, storage conditions, and assay methods. (4) To validate the results with more than one assay using different methodologies is usually necessary for EBC analysis. (5) Because many of the nonvolatile components found in EBC are identified by assays at their lower limits of accuracy, the use of lyophilization, dehydration, or freeze drying of the EBC coupled with resuspension in small volumes of highly pure water can improve the detection sensitivity. (6) EBC is highly dilute, assay controls must be performed appropriately with similar EBC component concentration scales. The following sections describe the most studied mediators in EBC. 

### 3.1. EBC pH

Respiratory symptoms such as cough, wheeze, dyspnea, and apnea are induced when acids are introduced into the airways or when the endogenous airway pH homeostasis is altered by diverse pulmonary diseases. The regulation of airway pH is involved in innate host defenses but also contributes to the pathophysiology of obstructive lung disease [14]. Therefore, it is important and beneficial to precisely and conveniently measure the airway pH in the diagnosis of many pulmonary conditions. Measurement of EBC pH or airway acidification is very challenging and complicated by poor reproducibility [15, 16]. The pH of raw EBC samples is unstable and is profoundly affected by carbon dioxide, the major volatile component of EBC. One strategy is to deaerate EBC with an inert (carbon dioxide free) gas such as argon or nitrogen to remove carbon dioxide. However, even after 20 min of deaeration, EBC samples may still contain an unpredictable amount of carbon dioxide, which may bias pH readings. To improve the reproducibility of pH readings and standardize the carbon dioxide effect on EBC pH, a carbon dioxide gas standardization method was developed [17, 18]. In this method, carbon dioxide is bubbled into an EBC sample for short intervals (1 s each) which cause a rapid but stepwise increase of the carbon dioxide partial pressure in the EBC sample. After each bubbling period, EBC pH and carbon dioxide partial pressure are measured simultaneously using a blood gas analyzer. A correlation plot between the EBC pH and carbon dioxide partial pressure is then generated. This correlation allows the calculation of pH at a carbon dioxide partial pressure of 5.33 kPa, the physiological alveolar carbon dioxide partial pressure. Although more reliable and convenient methods need to be developed for EBC pH measurement, this method currently provides the most reproducible EBC pH values. 

### 3.2. Arachidonic Acid Derivatives in the EBC

**Figure 2:**
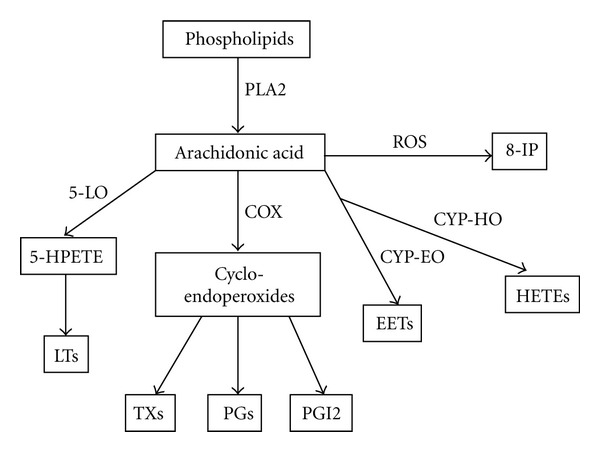
Metabolism of arachidonic acid. Arachidonic acid is released from phospholipids by the action of phospholipase A2 (PLA2). Arachidonic acid is metabolized by cyclooxygenases (COXs), lipoxygenases (LOXs), and cytochrome P450 (CYP). COXs metabolize arachidonic acid to prostaglandins (PGs), prostacyclin (PGI2), and thromboxanes (TXs). Leukotrienes (LTs) are the final arachidonic acid metabolites in the 5-lipoxygenase- (5-LO-) mediated pathway. CYP epoxygenases (CYP-EO) metabolize arachidonic acid to epoxyeicosatrienoic acid (EETs), and CYP hydroxylases (CYP-HO) metabolize arachidonic acid to hydroxyeicosatetraenoic acids (HETEs). 8-Isoprostane (8-IP) can be generated *in vivo* by the free radical-catalyzed peroxidation of arachidonic acid.

Arachidonic acid (AA) is a polyunsaturated omega-6 fatty acid present in the phospholipids of cell membranes. Arachidonic acid is released by the activation of the enzyme phospholipase A2 (PLA2) but can be further metabolized by cyclooxygenases (COX), 5-lipoxygenases (5-LO) and cytochrome P450 (CYP) [19–22]. A detailed scheme is presented in Figure 2 for arachidonic acid metabolism, where intracellular interactions control arachidonic acid conversion and activity. Cyclooxygenases generate prostanoids which can be further subdivided into three main groups: the prostaglandins (PGs), prostacyclin (PGI2), and thromboxanes (TXs), each of which is involved in some aspect of the inflammatory response. Arachidonate 5-lipoxygenase converts AA to yield leukotrienes (LTs). CYP epoxygenases (CYP-EO) convert arachidonic acid to epoxyeicosatrienoic acids (EETs). CYP hydroxylases (CYP-HO) metabolize arachidonic acid to hydroxyeicosatetraenoic acids (HETEs). Airway epithelial cells are sensitive to arachidonate metabolites and have abundant arachidonic acid and novel cyclooxygenases and lipoxygenases at increased levels relative to other cell types [23]. However, arachidonate metabolites can be synthesized by and have potent biologic effects on other airway cells such as leukocytes, smooth muscle, nerves, mucus glands, and platelets. 8-Isoprostane (8-IP), a prostaglandin (PG)-F2-like compound, belongs to the F2 isoprostane class that is produced *in vivo* by the free radical-catalyzed peroxidation of arachidonic acid [24]. Because of the transcellular feature of arachidonic acid metabolism and function, airway lining fluid is the critical medium for these actions. Significant amounts of arachidonic acid and its derivatives are present in ALF. 8-Isoprostane, LTs, and prostanoids have been detected in EBC and used as biomarkers for oxidative stress and respiratory infection. Methods used to detect arachidonic acid derivatives in the EBC include gas chromatography/mass spectrometry (GC/MS), liquid chromatography/mass spectrometry (LC/MS), radioimmunoassay (RIA), and enzyme immunoassay (EIA). 

Most studies measuring 8-IP used commercial EIA kits with a detection limit of 1 pg/mL [25]. GC/MS is a more sensitive method for 8-IP detection and has been used to validate EIA results [26]. 8-IP levels in the EBC of healthy subjects were reported in the range of 0–40 pg/mL. Increased concentrations of 8-IP in the EBC as a marker of oxidative stress has been demonstrated in multiple lung diseases, such as asthma [27, 28], COPD [29, 30], interstitial lung disease [31], and cystic fibrosis [32, 33]. LTs can also be measured in the EBC by EIA with a detection limit of 4 pg/mL. Other methods such as LC/MS/MS, GC/MS, and high-performance liquid chromatography (HPLC) are also used in LT detection [34]. LTs in EBC samples from healthy subjects range from 0 to 25 pg/mL. Elevated LTs were found to be correlated with parameters of inflammation in the lungs [35]. Similarly, prostanoids can be measured by EIA, RIA, and chromatographic techniques and are present in the range of 0–200 pg/mL in EBC [26, 36].

### 3.3. Oxygen and Nitrogen Reactive Species and Redox-Relevant Molecules in EBC

**Figure 3:**
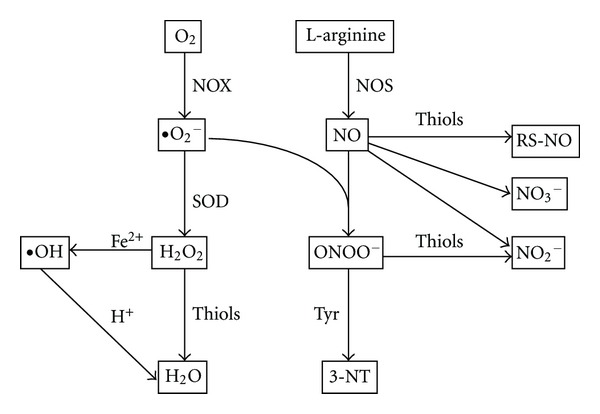
Reactive oxygen and nitrogen species and redox relevant molecules in EBC. Exhaled nitric oxide (NO) is derived from L-arginine by enzyme nitric oxide synthase (NOS). NO can combine with superoxide (•O_2_^−^) to form peroxynitrite (ONOO^−^). ONOO^−^ induces nitrosation of tyrosine (Tyr) residues and forms 3-nitrotyrosine (3-NT). NO can also react with thiols to form S-nitroso thiols (RS-NO). The end-products of NO are nitrite (NO_2_^−^) or nitrate (NO_3_^−^). •O_2_^−^ is one of major reactive oxygen species generated from NADPH oxidase (NOX) or mitochondrial electron transfer chain. •O_2_^−^ is converted to hydrogen peroxide (H_2_O_2_) by superoxide dismutases (SOD). H_2_O_2_ can be converted to the highly reactive hydroxyl radical (•OH), which is catalyzed by Fe^2+^ (Fenton reaction). H_2_O_2_ can be removed by thiol-specific antioxidant enzymes to form water.

Investigations of reactive oxygen species (ROS) and reactive nitrogen species (RNS) are among the interests of EBC biomarkers in many lung disease studies. Multiple RNS formation starts with nitric oxide (NO). NO is a volatile component of the EBC [3, 13, 37, 38] and is synthesized from the amino acid L-arginine by nitric oxide synthase (NOS) (Figure 3). Different cell types within the respiratory tract have been identified to contain NOS, including airway and alveolar epithelial cells, macrophages, neutrophils, eosinophils, mast cells, and vascular endothelial and smooth muscle cells. Superoxide anion (•O_2_^−^) is a ROS that reacts quickly with NO, to form highly reactive peroxynitrite (ONOO^−^). ONOO^−^ can cause the nitrosation of either tyrosine or tyrosine residues in proteins to form 3-nitrotyrosine (3-NT). Nitrotyrosine can be measured by enzyme immune assays or HPLC and MS [39–41]. NO can also react with thiols, such as cysteine, glutathione, or protein thiol residues to produce S-nitrosothiols (RS-NO) which can be measured by the colorimetric assay [42]. The end-products of NO metabolism are nitrite (NO_2_^−^) and nitrate (NO_3_^−^). In EBC, nitrite and nitrate can be measured by colorimetric, fluorometric, and chemiluminescent assays, or by ion, gas, and liquid chromatography [43, 44]. 

Hydrogen peroxide (H_2_O_2_) is another volatile molecule in EBC [3, 13]. In several cell types, H_2_O_2_ can be produced by superoxide dismutase (SOD) through conversion of the superoxide anion (•O_2_^−^). H_2_O_2_ can be released from both inflammatory and structural cells including neutrophils, eosinophils, macrophages, and epithelial cells. Since H_2_O_2_ is unstable in the EBC, samples should be freshly collected or rapidly frozen after collection. Common methods used to measure H_2_O_2_ include spectrophotometric, fluorometric, or chemiluminescent assays and indicate a concentration of ~200 nM in different pulmonary pathologies [45, 46]. Reactive oxygen species can degrade polyunsaturated lipids and form malondialdehyde (MDA), another biomarker of oxidative stress [47, 48]. The MDA present in the EBC can be measured by HPLC in the 10 nM concentration range [49, 50]. 

Increasing reactive oxygen and nitrogen species or their derivatives in the EBC are used as indicators of oxidative stress or inflammation in the respiratory track. Compared with healthy nonsmokers, concentrations of H_2_O_2_, MDA, RS-NO, 3-NT, NO_2_^−^, and NO_3_^−^ are increased in the EBC of patients with asthma, COPD, idiopathic pulmonary fibrosis, and cystic fibrosis [1, 3, 13, 47]. In addition to ROS/RNS, the ALF also contains significant antioxidant compounds such as cysteine (Cys) and glutathione (GSH). Although the GSH concentration in the bronchoalveolar lining fluid is in the magnitude of *μ*M, the GSH concentration in the EBC is in the magnitude of nM resulting in a 1000 dilution of GSH in the EBC pool when compared to the bronchoalveolar lavage fluid [51–53]. When subjects with or without an alcohol use disorder were compared, both the lavage fluid and the EBC demonstrated ~80% decrease in GSH and oxidation of the thiol/disulfide redox potential by ~40 mV [54]. This suggests that changes in the EBC can be representative of physiological changes in the ALF. 

### 3.4. EBC Proteins

Playing central roles in both the immunity and inflammation aspects of the host defense system, cytokines can be classified by their ability to promote or inhibit inflammatory response: proinflammatory cytokines (IL-1*β*, IL-2, IL-6, IL-8, IL-12, IL-17, IFN-*γ*, and TNF-*α*), anti-inflammatory cytokines (IL-4, IL-5, IL-10, IL-13, and TGF-*β*), and chemokines (IL-8, MCP-1, and MIP-1ß). Cytokines can also be grouped based on the type of T-lymphocytes with which they are associated. T helper (Th) lymphocytes stem from T CD4+ lymphocytes precursors (Th0), and depending on the cytokine environment, helper T cells can differentiate into three major different phenotypes: Th1, Th2, and Th17. The Th1 cytokine profile includes IFN-*γ*, TNF-*α*, IL-1, IL-2, and IL-12. The Th2 cytokines are IL-4, IL-5, IL-6, IL-10, and IL-13. Th17 cytokines (IL-17, IL-21, IL-22, TNF-*α*, and TGF-*β*) include “regulatory” cytokines involved in the immune tolerance process. Systematic cytokine profiling is useful in diagnosis and therapeutic treatment for airway diseases. Identification of cytokines in the EBC using ELISA assays has been reported. In the EBC, the cytokines IL-1*β*, IL-2, IL-4, IL-5, IL-6, IL-8, IL-10, IL-17, IFN-*γ*, TGF-*β*, and TNF-*α* have been reported to be in the ~50 pg/mL range [55–57]. An EBC dilution factor of 10^−3^  is generally accepted relative to ALF [9, 58] giving an estimated ALF cytokine level in the order of 50 ng/mL. However, cytokine detection in EBC is often at the lower limits of detection for the assay, and these values are further complicated by the absence of a gold standard for dilution of the EBC or the bronchoalveolar lavage. 

Due to the complications from detection bias and correction for dilution, the measurement of multiple substances concurrently and determination of their ratios would reduce the detection bias and avoid artifacts due to correction for dilution. For cytokine analysis, a shift in the Th1/Th2 ratio usually accompanies with varied immune response in pathological pulmonary conditions. Examples of such approach have been reported in determining the IFN-*γ*(Th1)/IL-4(Th2) ratio [56, 59]. Systematic approaches, such as proteomic analysis of EBC, have been previously used and may provide a more detailed overall view about cytokine profile in the EBC. However, EBC is challenging for proteomics studies because of low protein concentrations. Proteome analysis of low-abundance proteins depends on the complexity of the protein mixture, the power of the resolution, and the sensitivity of the separation and identification methods. Although proteomic analysis has been used with EBC, the majority of the proteins detected were keratins, a family of fibrous structural proteins present in the outer layer of human skin [60–63]. Although keratin content in EBC has shown significant differences between disease subjects and healthy control groups [61, 62, 64], the EBC cytokine profile is still a useful tool for monitoring lung inflammation. To detect low-abundance EBC cytokines present in the pg/mL range, advanced techniques such as immunoaffinity depletion and selective target enrichment are required for proteomic analysis [65, 66]. 

## 4. Conclusion

EBC is an exciting new approach for investigating lung diseases because it is noninvasive and contains many potential biomarkers. However, the key limitation for the EBC as a diagnostic tool is the low concentration range of different EBC biomarkers. Currently, efforts to address methodological issues include standardization of sample collection and validation of analytical techniques. To establish the reproductively of EBC measurements, more sensitive assays and new molecular detection techniques are necessary. Metabolomics and proteomics can provide systemic profile for EBC biomarkers and may prove to be useful in screening and diagnosing lung diseases. In addition, systematic techniques that can concurrently measure multiple EBC substances may limit detection bias and provide patterns of biomarkers that are sensitive to disease and disease treatments.
